# The Evolution of Rumors on a Closed Social Networking Platform During COVID-19: Algorithm Development and Content Study

**DOI:** 10.2196/30467

**Published:** 2021-11-23

**Authors:** Andrea W Wang, Jo-Yu Lan, Ming-Hung Wang, Chihhao Yu

**Affiliations:** 1 Information Operations Research Group Taipei Taiwan; 2 Department of Information Engineering and Computer Science Feng Chia University Taichung Taiwan; 3 Department of Computer Science and Information Engineering National Chung Cheng University Chiayi Taiwan

**Keywords:** COVID-19, rumors, rumor diffusion, rumor propagation, social listening, infodemic, social media, closed platform, natural language processing, machine learning, unsupervised learning, computers and society

## Abstract

**Background:**

In 2020, the COVID-19 pandemic put the world in a crisis regarding both physical and psychological health. Simultaneously, a myriad of unverified information flowed on social media and online outlets. The situation was so severe that the World Health Organization identified it as an infodemic in February 2020.

**Objective:**

The aim of this study was to examine the propagation patterns and textual transformation of COVID-19–related rumors on a closed social media platform.

**Methods:**

We obtained a data set of suspicious text messages collected on Taiwan’s most popular instant messaging platform, LINE, between January and July 2020. We proposed a classification-based clustering algorithm that could efficiently cluster messages into groups, with each group representing a rumor. For ease of understanding, a group is referred to as a “rumor group.” Messages in a rumor group could be identical or could have limited textual differences between them. Therefore, each message in a rumor group is a form of the rumor.

**Results:**

A total of 936 rumor groups with at least 10 messages each were discovered among 114,124 text messages collected from LINE. Among 936 rumors, 396 (42.3%) were related to COVID-19. Of the 396 COVID-19–related rumors, 134 (33.8%) had been fact-checked by the International Fact-Checking Network–certified agencies in Taiwan and determined to be false or misleading. By studying the prevalence of simplified Chinese characters or phrases in the messages that originated in China, we found that COVID-19–related messages, compared to non–COVID-19–related messages, were more likely to have been written by non-Taiwanese users. The association was statistically significant, with *P*<.001, as determined by the chi-square independence test. The qualitative investigations of the three most popular COVID-19 rumors revealed that key authoritative figures, mostly medical personnel, were often misquoted in the messages. In addition, these rumors resurfaced multiple times after being fact-checked, usually preceded by major societal events or textual transformations.

**Conclusions:**

To fight the infodemic, it is crucial that we first understand why and how a rumor becomes popular. While social media has given rise to an unprecedented number of unverified rumors, it also provides a unique opportunity for us to study the propagation of rumors and their interactions with society. Therefore, we must put more effort into these areas.

## Introduction

Online social media has democratized content. By creating a direct path from content producers to consumers, the power of production and sharing of information has been redistributed from limited parties to general populations. However, social media platforms have also given rise to the proliferation of misinformation and enabled the fast dissemination of unverified rumors [1-3]. In 2020, the COVID-19 pandemic put the world in a crisis regarding both physical and psychological health. A myriad of unverified information flowed on social media. Rumors and claims of erroneous health practices even interfered with the control of COVID-19 in various parts of the world [4,5]. The World Health Organization (WHO) identified this situation as an infodemic in February 2020 [6], indicating its seriousness.

Previous studies revealed that people relied on social media to gather COVID-19 information and guidelines [7,8]. Efforts have, thus, been put into studies examining true and false rumors on social media [9-11]. For example, Cinelli et al [9] compared feedback to reliable and questionable COVID-19 information across five platforms, including Twitter, YouTube, and Gab. Gallotti et al [10] looked at how much unreliable COVID-19 information Twitter users were exposed to across countries.

Machine learning and deep learning techniques have been employed to study COVID-19 posts on social media, with much of the focus on topic modeling, sentiment analysis, and misinformation detection [12-25]. Both sentiment analysis and misinformation detection are supervised classification problems. Many studies have employed the Valence Aware Dictionary and Sentiment Reasoner (VADER) model or long short-term memory (LSTM) for sentiment analysis and ensemble machine learning models, such as Extreme Gradient Boosting (XGBoost), for misinformation detection [13-15,17-19,23,25]. Topic modeling, on the other hand, is an unsupervised clustering method. Among topic modeling studies, latent Dirichlet allocation (LDA) was the most widely used algorithm [12,15,17,19,21,22,24], and other favorites included k-means clustering [14,16]. For example, Chandrasekaran et al [15] utilized LDA to extract 26 topics among 13.9 million English COVID-19 Twitter posts. Then they adopted the VADER model to compute sentiment scores for each topic. Jelodar et al [19] employed LDA to extract topics from 560,000 COVID-19 Twitter posts and then used the LSTM neural network to identify the sentiments of the posts. Kwok et al [21] employed LDA to extract topics and Stanford University’s CoreNLP (natural language processing) to study the sentiments of Twitter posts regarding COVID-19 vaccinations from Australian Twitter accounts. Also, Chen et al [16] compared the COVID-19 discussions on Twitter and Weibo using t-distributed stochastic neighbor embedding dimensionality reduction with the k-means clustering algorithm to extract topics.

Despite the instructive knowledge provided by the aforementioned machine learning studies, there are two identifiable gaps. First, most studies concentrated on *public* social media platforms, with the majority using Twitter as the data source [12-19,21,22,24,25]. Investigations on closed social media platforms, such as WhatsApp, WeChat, Telegram, or LINE, remain extremely scarce. Secondly, most studies looked at posts via their high-level theme, such as “misconceptions and complaints about COVID-19 control” [21], “psychological stress” [17], or “government response” [15]. There were limited efforts put into the study of individual narratives or rumors under a high-level theme, for example, rumors such as “protect yourself from coronavirus by putting bleach in your body” and “check for COVID-19 by holding your breath for 10 seconds or longer” under the theme of “erroneous health practices.”

While high-level themes and sentiments can give us an overview of the public discourse, the capability to efficiently identify individual narratives would be extremely helpful for picking up trending rumors and claims. Discussions on social media platforms are most likely not independent from each other. Thus, simply looking at billions of individual messages is not effective for identifying what rumors are receiving attention. Therefore, there is an apparent need for an efficient way to group and extract the narratives to recognize the popular ones.

**Figure 1:**
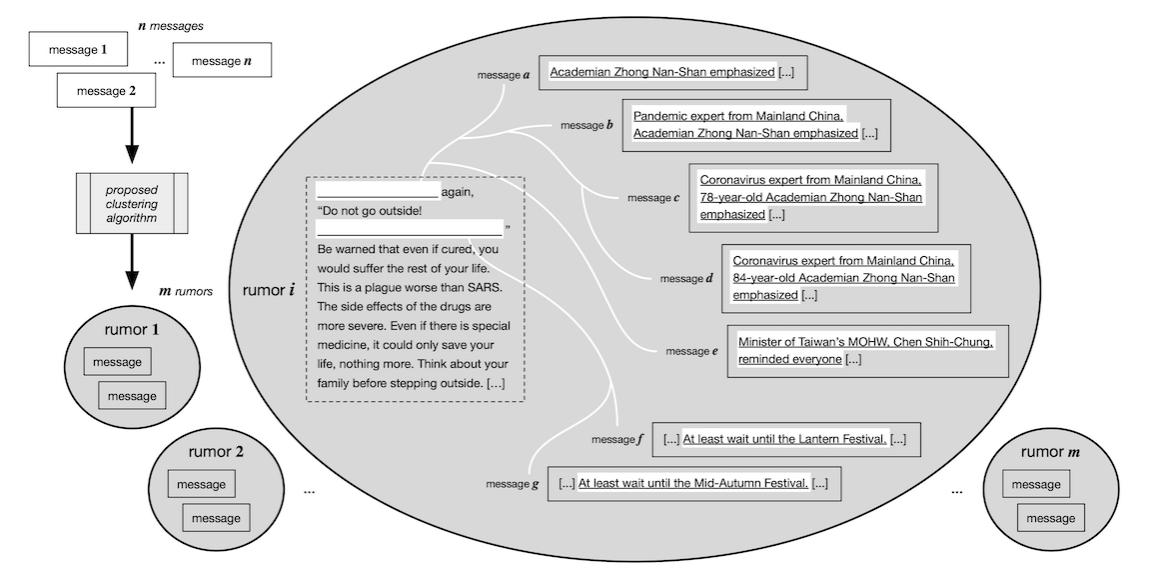
A graphical depiction of this study's goal in using machine learning to extract individual rumors from a pool of social media messages. MOHW: Ministry of Health and Welfare.

Recognizing the limitations from previous studies and to solve the aforementioned problem, our goal was to use machine learning to identify individual COVID-19 rumors from a pool of social media messages, as shown in Figure 1. After identifying the rumors, we then investigated the propagation patterns and textual transformation of those rumors on a closed platform. To achieve this, we proposed a classification-based clustering algorithm to efficiently group tens of thousands of messages according to the similarity of messages. Then, we applied the algorithm to the suspicious messages on LINE, a popular messaging platform in Taiwan. Furthermore, according to the clustering results, we investigated how the messages evolved from temporal and cultural perspectives during the pandemic. To the best of our knowledge, this is the first study to examine COVID-19 rumor diffusion on a closed platform.

## Methods

### Data Collection

LINE is an instant messaging platform. According to the 2018 Taiwan Communication Survey (TCS), 98.5% of people in Taiwan used LINE as their primary messaging tool [26], making it the most popular closed messaging platform. In light of the increasing amount of unreliable information being exchanged through LINE, fact-checking agencies or groups, such as the Taiwan FactCheck Center, Cofacts, or MyGoPen, have developed LINE chatbots for users to voluntarily forward suspicious messages. These chatbots archive the messages and check them against their existing databases to reply with the fact-checked results.

We obtained a data set of suspicious messages forwarded by LINE users to a fact-checking LINE bot between January and July 2020. The data set included messages related to COVID-19 as well as other topics.

Along with the text content of each reported message, we also obtained the report time of each message and a unique identifier for the LINE user that reported the message. The user identifiers we received were scrambled; therefore, it was not possible for us to use the identifiers to attribute any reported message back to any actual LINE user.

### Data Preprocessing

After obtaining the text messages, we preprocessed them using the following steps. First, we removed all characters that were neither simplified Chinese nor traditional Chinese. Second, we tokenized each message using the Jieba library [27] in Python (version 3.7; Python Software Foundation) and then removed tokens that were Chinese stop words from the token list. To focus on longer messages, we only kept messages with at least 20 tokens from our data set. Finally, the CountVectorizer module from Python’s scikit-learn package [28] was used to create a binary word vector for each message.

### Clustering Messages Into Rumor Groups by the Classification-Based Clustering Algorithm

In order to determine what messages belonged to the same rumor, we needed to define distance between messages. We wanted two messages, A and B, to be close to each other if the overlapping text between the two constituted the majority of both messages. When the overlapping text makes up the majority of A but not B, it signals that message A only constitutes a portion of B, meaning that B is likely a combination of several other rumors. In this situation, A and B should be in different groups; therefore, we would like the distance between them to be larger. Based on this idea, we defined the distance between two messages, A and B, to be as follows:

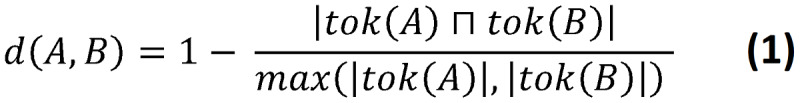



where *tok*(·) is the set of tokens of one message and |·| denotes the number of elements in a set.

While most work relied on the LDA or k-means algorithm to separate messages into groups, both algorithms required a predefined number of final groups. That is, the users need to tell the algorithm how many groups to separate the messages into before being applied. Even though what we wanted to discover was how many narratives, or rumors, there were in all the messages by comparing the distance (equation 1) among all messages, such a requirement contradicted our needs. Hierarchical agglomerative clustering (HAC), on the other hand, starts by merging messages closer to one another into clusters and then iteratively merging closer clusters together until the distance between each cluster exceeds a predefined threshold. That is, instead of predefining a specific number of final groups like in LDA or k-means clustering, HAC determines the number of groups based on a predefined distance threshold. In addition, HAC has the advantage of accepting self-defined distance metrics. Therefore, HAC was the clustering algorithm that fitted our needs.

**Figure 2:**
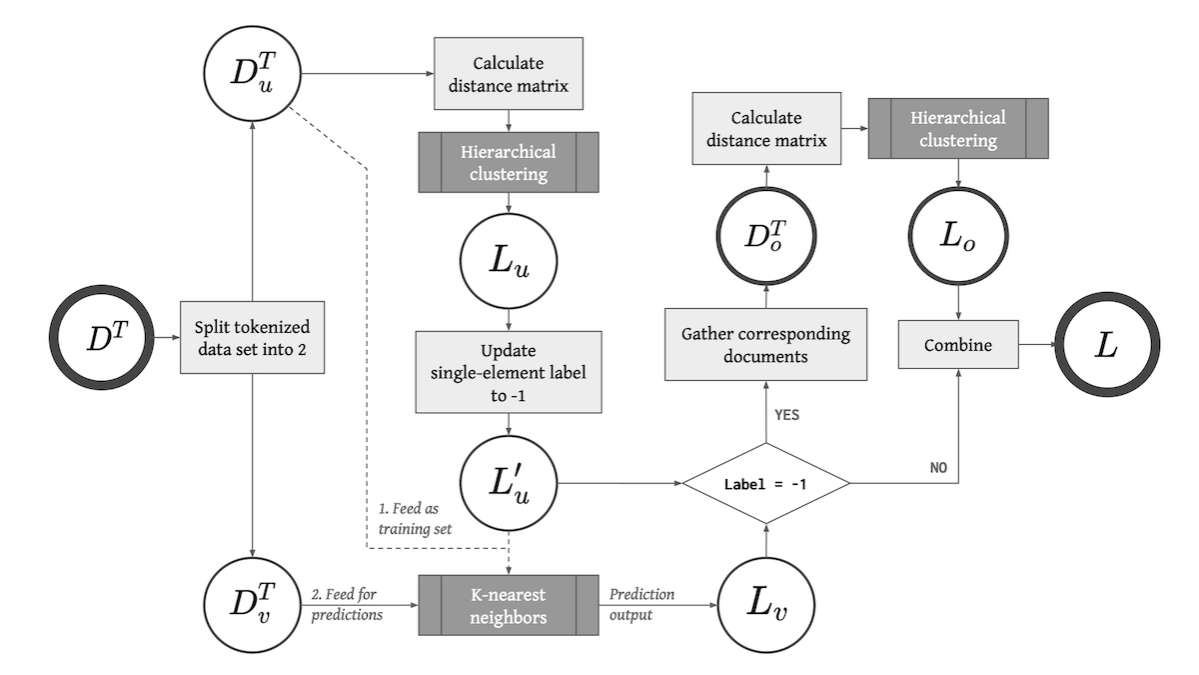
Flowchart for the classification-based clustering algorithm (hierarchical agglomerative clustering + k-nearest neighbors algorithm).

However, HAC can be quite slow and memory consuming. It suffers with large data sets, especially in the case of social media messages. Therefore, we devised a classification-based clustering algorithm, one that combined the k-nearest neighbors (KNN) algorithm with HAC, to efficiently perform the clustering task. The idea was to randomly select a portion of messages on which to perform HAC; the result was then used to train a KNN algorithm. The trained KNN algorithm was subsequently used to predict the rest of the messages. A detailed algorithm is outlined in Textbox 1, and a flowchart of the algorithm is presented in Figure 2. The experimentation details are outlined in Multimedia Appendix 1, and we demonstrate the efficiency and effectiveness of this algorithm in the following subsection. The algorithm was implemented with the KNeighborsClassifiers and AgglomerativeClustering modules from the Python library scikit-learn [28]. The library gensim (version 3.8.3) [29] was also used in experiments to implement the LDA model for comparisons. We released the code to implement the model in a GitHub repository [30].

**The classification-based clustering algorithm (hierarchical agglomerative clustering plus k-nearest neighbors algorithm).**

**Notation:**

*(A)_j_*: *j*^th^ element of set *A*.

**Input:**

1. *D*: the set of all documents to be grouped.
2. *D^T^*: the set of tokenized documents. The order is preserved as *D*.
3. Train portion *u*: a number >0 and ≤1.
4. Distance threshold λ: a number >0 and ≤1. Throughout this paper, we set λ=0.6.

**Algorithm:**

1. Select *u* × |*D^T^*| elements from *D^T^*, denoted as *D^T^_u_*, and the rest not selected as set *D^T^_v_*.
2. Construct distance matrix *M* for *D^T^_u_*, where *M_ij_* = *d*((*D^T^_u_*)*_i_*, (*D^T^_u_*)*_j_*) by equation 1. Note that *M* is symmetric.
3. Feed *M* into hierarchical clustering with a distance threshold of λ. We will get back a sequence of labels *L_u_*, where (*L_u_*)*_i_* is the label of element (*D^T^_u_*)*_i_*. Elements with the same label are in the same cluster. Since the label itself does not carry meaning, manipulate them so they are all nonnegative whole numbers.
4. For each unique label *x* in *L_u_*, if |{ *k* | *k* = *x* ∀ *k* ∈ *L_u_* }| = 1, then replace the value of *x* with −1. Denote the updated label set as *L’_u_*.
5. Train a k-nearest neighbors classifier *K* using the training set (*D^T^_u_*, *L’_u_*). Then use *K* to predict the labels of *D^T^_v_*. Denote the prediction as *L_v_*.
6. Construct *L* by combining *L’_u_* and *L_v_*, where (*L*)*_i_* is the label of (*D^T^*)*_i_*.
7. Construct *D^T^_o_* = {*d_i_* | Label (*d_i_*) = –1 ∀ *d_i_* ∈ *D^T^*}.
8. Redo steps 2 and 3 for *D^T^_o_*. Denote the output as *L_o_*. Make sure the values of *L_o_* do not overlap with the values of *L* from step 6.
9. Update *L* from step 6 with *L_o_*.

**Algorithm output:**

A list of labels *L*, where (*L*)*_i_* denotes the label of document (*D*)*_i_*. Note that the value of the label itself does not carry any meaning. However, elements in *D^T^* with the same label belong to the same group.

### Comparing the Classification-Based Clustering Algorithm With Other Popular Algorithms

**Figure 3:**
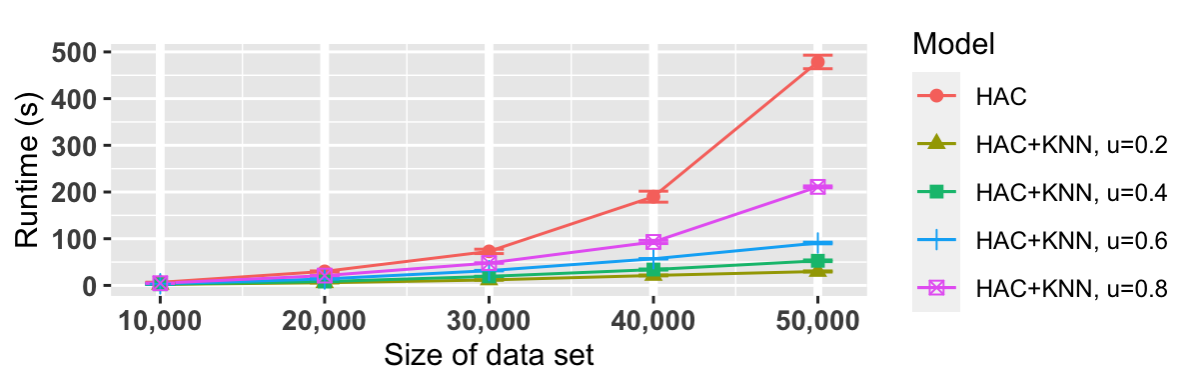
Speed comparison with 95% CIs between HAC and HAC+KNN across different levels of train portion *u*. HAC: hierarchical agglomerative clustering; KNN: k-nearest neighbors.

**Figure 4:**
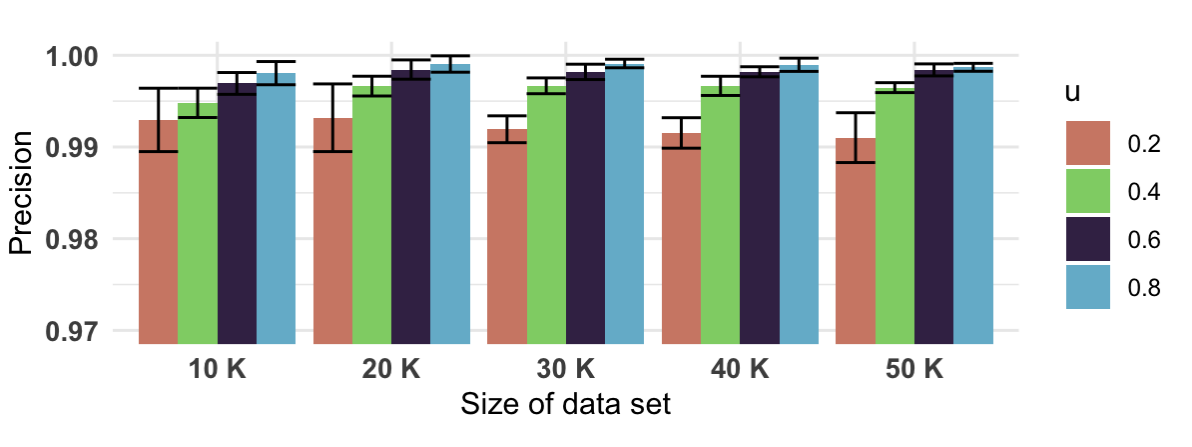
Precision values and 95% CIs (whiskers) of the HAC+KNN algorithm across different data set sizes and train portion *u*. HAC: hierarchical agglomerative clustering; KNN: k-nearest neighbors.

**Figure 5:**
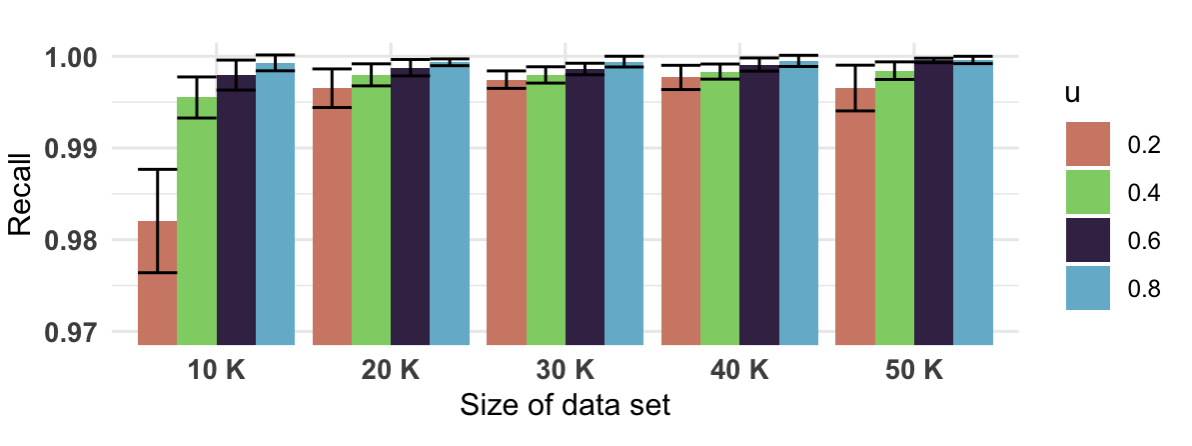
Recall values and 95% CIs (whiskers) of the HAC+KNN algorithm across different data set sizes and train portion *u*. HAC: hierarchical agglomerative clustering; KNN: k-nearest neighbors.

**Figure 6:**
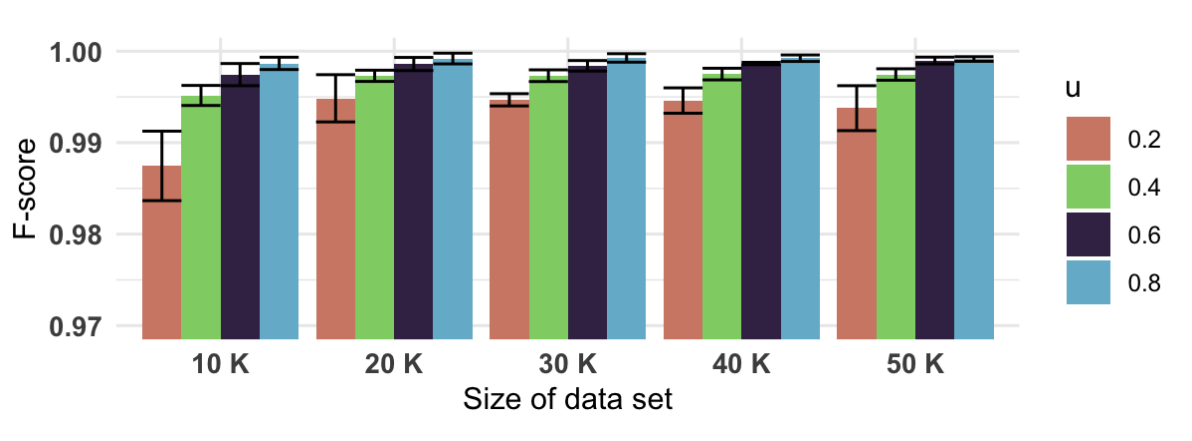
*F* scores and 95% CIs (whiskers) of the HAC+KNN algorithm across different data set sizes and train portion *u*. HAC: hierarchical agglomerative clustering; KNN: k-nearest neighbors.

From Figure 3, we can see that the classification-based clustering algorithm, the HAC+KNN model, greatly reduced the runtime compared to using only HAC, especially when the train portion value *u* was less than 0.60. Furthermore, such a significant gain in speed did not compromise the clustering results. With the HAC model’s results as the gold standard to compare with, the precision values (Figure 4), recall values (Figure 5), and *F* scores (Figure 6) from the HAC+KNN model remained greater than 99% when the train portion *u* was not lower than 0.40. The results demonstrated that the HAC+KNN model’s assignments of groups were complete, as measured by recall, and the use of KNN did not introduce too many errors in each group, as measured by precision.

We observed that the runtime of the k-means clustering was 10 times slower than that of the HAC algorithm, and the LDA model’s runtime was the slowest among all models (Table 1). In addition, the precision of the LDA model was very low, meaning that predicted groups had many false positives. While the precision of the k-means model was comparable to that of the HAC+KNN model, recall was only 73%. This showed that the k-means model missed out on many messages.

**Table 1:** Performance comparisons between HAC, HAC+KNN, LDA, and k-means models for data sets with 10,000 messages.

Model	Runtime (seconds), mean (SD)	Precision, mean (SD)	Recall, mean (SD)	*F* score, mean (SD)
HAC^a^	6.594 (0.245)	N/A^b^	N/A	N/A
HAC+KNN^c^ (*u*=0.2)	2.172 (0.097)	0.993 (0.003)	0.982 (0.005)	0.986 (0.004)
HAC+KNN (*u*=0.4)	2.502 (0.023)	0.995 (0.001)	0.996 (0.002)	0.995 (0.001)
HAC+KNN (*u*=0.6)	3.418 (0.071)	0.997 (0.001)	0.998 (0.001)	0.997 (0.001)
HAC+KNN (*u*=0.8)	4.697 (0.146)	0.998 (0.001)	0.999 (0.001)	0.999 (0.001)
LDA^d^	1788.981 (62.444)	0.624 (0.029)	0.939 (0.006)	0.704 (0.023)
K-means	41.143 (1.334)	0.993 (0.002)	0.734 (0.011)	0.823 (0.010)

^a^HAC: hierarchical agglomerative clustering.

^b^N/A: not applicable, because model does not include the parameter *u*.

^c^KNN: k-nearest neighbors.

^d^LDA: latent Dirichlet allocation.

### Determining Whether a Rumor Is Related to COVID-19

A rumor group contains many messages. To determine if a rumor group is related to COVID-19, we first identified how many messages in the group contained any of the COVID-19 keywords from the list that we put together (Textbox 2). Next, rumor groups with more than 60% of messages containing COVID-19–related keywords were passed to the authors to decide if such a rumor was really about COVID-19. If a rumor was deemed COVID-19–related, then all messages in the group were also deemed COVID-19–related, regardless of whether that message itself contained the keywords. Recognizing COVID-19–relatedness by close neighbors of each message is a more inclusive approach, as there were messages without the keywords that were obviously related to the pandemic; see Multimedia Appendix 2 as an example.

**A list of 33 COVID-19–related keywords.**

指揮中心, 奎寧, 急性呼吸道感染, 新型病毒, 疫情, 口罩, 負壓, 抗疫, 陽性, 新型冠狀病毒, 潛伏期, 李文亮, 纖維化, 自主管理, 群聚, 隔離, 確診, 武漢, 譚德塞, 陰性, 新冠, 染疫, 武肺, 封城, 肺炎, 自主健康管理, 防疫, 冠狀, 家庭感染, covid, ibuprofen, 2019-ncov, coronavirus

## Results

### Data Set

**Figure 7:**
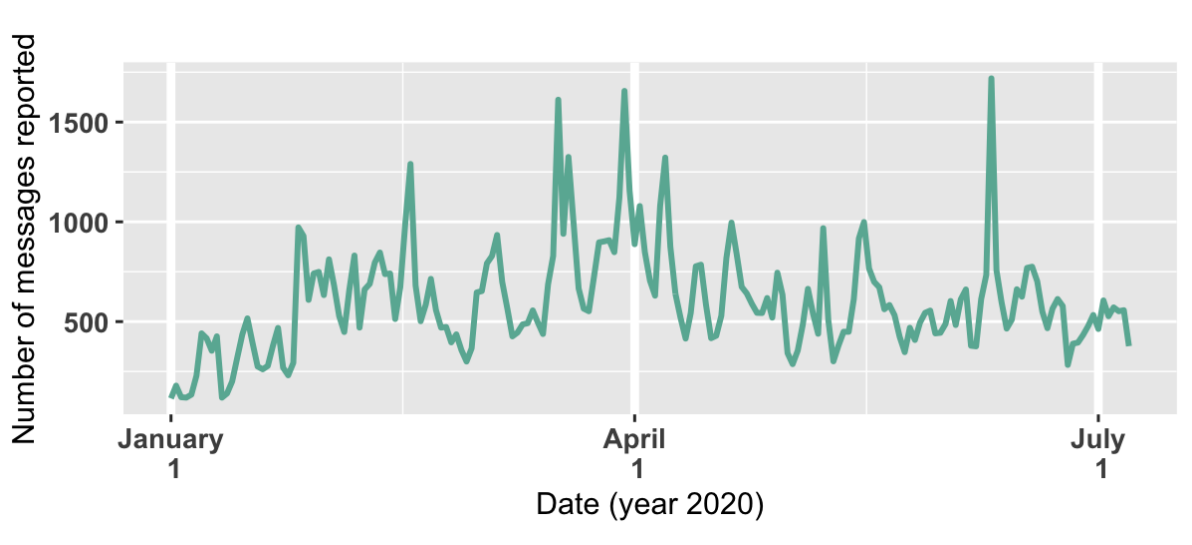
Distribution of 114,124 messages by report dates.

Our data set, after preprocessing, contained 114,124 messages. The character distribution is presented in Table 2, and the number of messages reported per date is shown in Figure 7.

**Table 2:** Breakdown of characters in the data set of 114,124 suspicious messages.

Statistic	Type of character				
	All	Chinese	Digit	Alphabetical	Others^a^
Minimum, n	24	24	0	0	0
Median (IQR)	233 (333)	145 (225)	7 (17)	2 (22)	38 (79)
Maximum, n	10,012	8132	3252	7014	5532

^a^This category includes characters such as punctuation marks and emojis.

### Rumor Group Overview

By using the HAC+KNN algorithm, 114,124 messages were separated into 12,260 rumor groups. A total of 8529 rumor groups had only 1 message. Therefore, the rest of the 105,595 messages were separated into 3731 rumor groups. There were 936 rumor groups with at least 10 messages, with the largest one having 2546 messages. We present the statistics of the rumor group sizes in Table 3.

**Table 3:** Statistics of the rumor group sizes.

Minimum number of messages per rumor group	Messages				
	Mean (SD)	Maximum, n	3rd quartile, n	2nd quartile, n	Total, n
1	9.309 (71)	2546	2	1	114,124
2	28.302 (126.907)	2546	10	3	105,595
10	102.96 (238.31)	2546	75	27	96,373

Among 936 rumor groups with at least 10 messages, we identified 396 (42.3%) that were related to COVID-19; these consisted of a total of 42,829 messages. Among 396 COVID-19–related rumor groups, 134 (33.8%) were deemed false or misleading by either the Taiwan FactCheck Center or MyGoPen, two International Fact-Checking Network (IFCN)–certified fact-checking agencies in Taiwan.

After recognizing many messages containing simplified Chinese characters or phrases originating from China, we compared the prevalence of those characters and phrases between COVID-19–related and non–COVID-19–related messages. Compared to non–COVID-19–related messages, the pool of COVID-19–related messages had significantly more messages using simplified Chinese characters or phrases that originated from China (Table 4). The association was significant as determined by the chi-square independence test with Yates’ continuity correction (χ^2^_1_=1088.0, n=96,373; *P*<.001).

**Table 4:** Contingency table of COVID-19–relatedness using simplified Chinese characters or phrases originating from China.

Message type	Messages with simplified Chinese characters or phrases originating from China, n		Total messages, n
	Yes	No	
Related to COVID-19	16,957	25,872	42,829
Not related to COVID-19	15,776	37,768	53,544
Total	32,733	63,640	96,373

**Figure 8:**
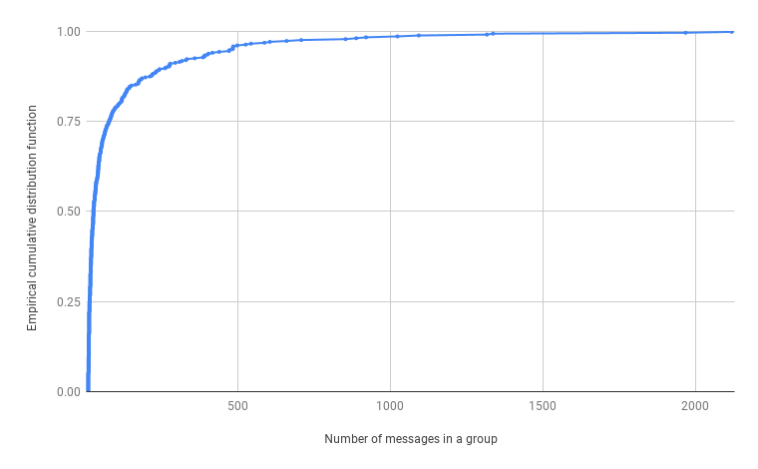
Empirical cumulative distribution function of the number of messages in COVID-19–related rumor groups.

The COVID-19–related rumor group sizes had a very long-tailed distribution (Figure 8). Most of the rumor groups only contained a few messages. In fact, only 15 rumor groups contained more than 1000 messages. In the following subsection, we discuss how we qualitatively analyzed the three COVID-19 rumor groups with the largest number of messages.

### Case Studies of the Three Largest COVID-19–Related Rumor Groups

#### Overview

We qualitatively analyzed the three rumor groups with the largest number of messages among the 936 COVID-19–related rumor groups. In fact, a total of 7523 messages from the three rumor groups made up 17.6% of all 42,829 COVID-19–related messages.

To study the interactions of the rumors’ popularity with society, we picked out some major societal events, as shown in Table 5. While there were multiple important events regarding the pandemic every day, we picked out incidents that were the first occurrences.

**Table 5:** Major societal events related to COVID-19.

Date (year 2020)	Events
February 9	First asymptomatic laboratory-confirmed COVID-19 case in Taiwan
February 15	First COVID-19 death case in Taiwan
February 21	Passengers on Diamond Princess cruise ship returned to Taiwan
March 11	COVID-19 declared a global pandemic by the World Health Organization
March 18	The director of the CECC^a^, Chen Shih-Chung, went to the Legislative Yuan for interpellation about the pandemic for the first time A total of 100 confirmed cases was reached; single-day confirmed cases hit record high for 3 consecutive days
March 26	The CECC released the first report on the analysis of confirmed cases in Taiwan
March 30	First death case in Taiwan’s first hospital cluster infection
April 1	The day before a 4-day long weekend First day of mask requirement on public transportation
April 5	The last day of a 4-day long weekend

^a^CECC: Central Epidemic Command Center.

#### Case 1: Do Not Go Outside!

**Figure 9:**
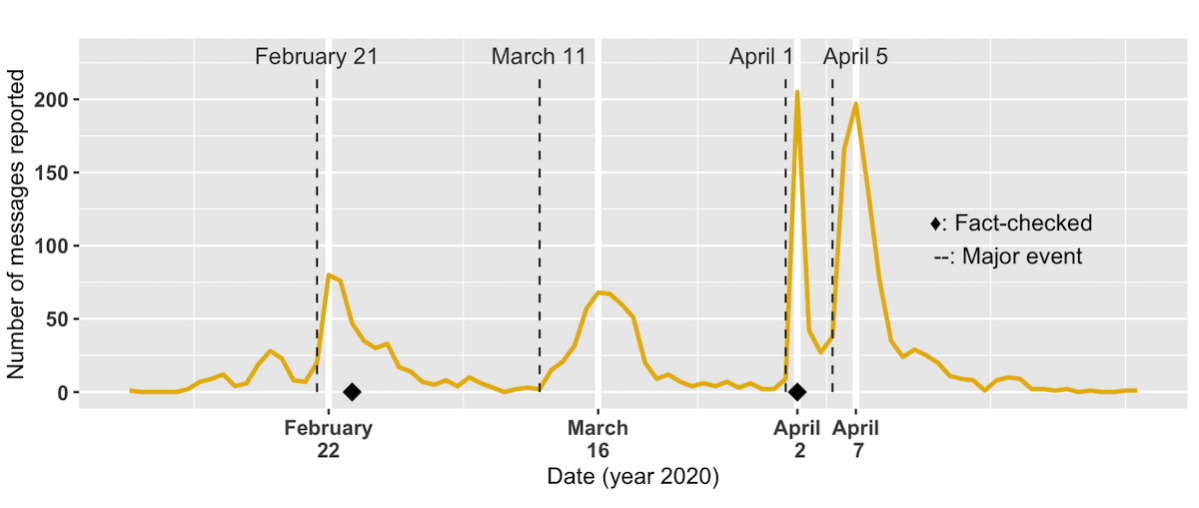
The number of Case 1 (ie, "Do not go outside!") messages reported by date. The number peaked on April 2 with 205 messages, when messages started misquoting the Central Epidemic Command Center (CECC) director. There were also a large number of reports after a 4-day long weekend on April 6 with 166 messages and on April 7 with 197 messages. Refer to Table 5 for major societal events.

The rumor content for Case 1 is presented in Textbox 3. This rumor first appeared in the data set on February 2, 2020. Over the course of 3.5 months, there were a total of 2119 messages reported. The reported messages went viral at least four times: they peaked on February 22 with 80 messages, they peaked on March 16 with 68 messages, they reached the highest number on April 2 with 205 messages, and they peaked on April 7 with 197 messages (Figure 9). During this period, we observed several content changes (Table 6).

**Content of Case 1 rumor.**

English translation:

Academian Zhong Nan-Shan emphasized again, “Do not go outside! At least wait until the Lantern Festival.” Be warned that even if cured, you would suffer the rest of your life. This is a plague worse than SARS. The side effects of the drugs are more severe. Even if there is special medicine, it could only save your life, nothing more. Think about your family before stepping outside...This is a war, not a game...No one is an outsider in this war...Please share it with others. By Zhong Nan-Shan.

Original content:

鐘南山院士再次強調：別出門，元宵後，再看疫情控制情況！警告：一旦染上，就算治癒了，後遺症也會拖累後半生！這場瘟疫比17年前的非典更嚴重，用的藥副作用更大。如果出了特效藥，也只能保命，僅此而已！出門前想想你的家人，別連累家人，能不出門就不出門，大家一起轉發吧！這是一場戰役，不是兒戲，收起你盲目的自信和僥倖心理，也收起你事不關己高高掛起的態度，在這場戰役中沒有局外人！在家！在家！在家！不要點贊！求轉發——鐘南山

**Table 6:** Change log for Case 1 rumor content.

Date (year 2020)	English translation (original)	
	Previous content	New content
February 17	Academian Zhong Nan-Shan emphasized (鍾南山院士再次強調)	Pandemic expert from Mainland China, Academian Zhong Nan-Shan emphasized (大陸防疫專家鍾南山院士再次強調)
February 18	Academian Zhong Nan-Shan emphasized (鍾南山院士再次強調)	Coronavirus expert from Mainland China, 78-year-old Academian Zhong Nan-Shan emphasized (大陸，冠狀病毒專家鐘南山78歲院士再次強調)
February 27	Academian Zhong Nan-Shan emphasized (鍾南山院士再次強調)	Coronavirus expert from Mainland China, 84-year-old Academian Zhong Nan-Shan emphasized (大陸，冠狀病毒專家鐘南山84歲院士再次強調)
April 1	Academian Zhong Nan-Shan emphasized (鍾南山院士再次強調)	Minister of Taiwan’s MOHW^a^, Chen Shih-Chung, reminded everyone (台灣衛福部長陳時中提醒大家)
February 18	Do not go outside! At least wait until the Lantern Festival. (別出門，元宵後，再看疫情控制情況)	Do not go outside! At least wait until the Dragon Boat Festival. (別出門，端午節過後，再看疫情控制情況)

^a^MOHW: Ministry of Health and Welfare.

First, the time-sensitive information in the messages evolved. In early February, most messages mentioned “Lantern Festival,” which took place on February 8, 2020. However, from February 18 onward, there were messages that replaced “Lantern Festival” with “March.” Then, after March 10, most messages included “Dragon Boat Festival,” which took place on June 25, 2020.

**Figure 10:**
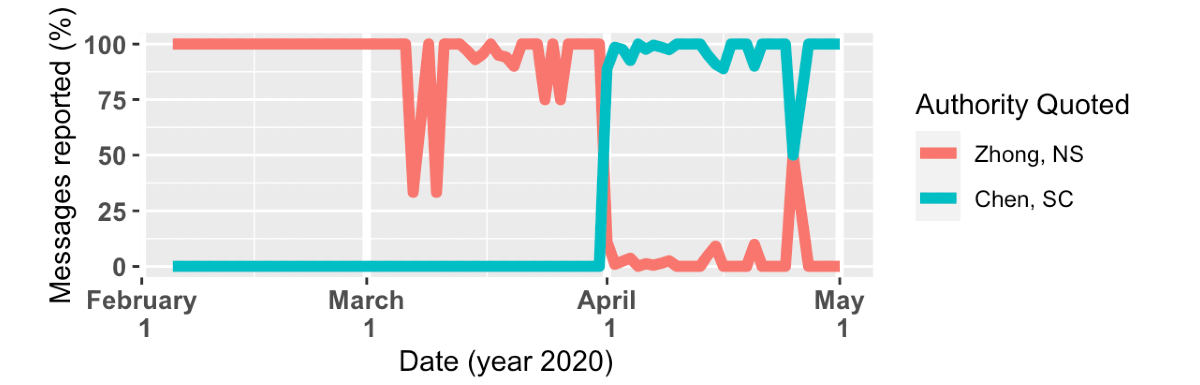
Chen Shih-Chung replaced Zhong Nan-Shan as the most quoted party in the Case 1 rumor after April 1, 2020.

Second, among 2119 reported messages, 2095 (98.9%) falsely quoted authority. Zhong Nan-Shan, the leader of China’s National Health Commission’s expert panel for investigating the COVID-19 outbreak in China, and Chen Shih-Chung, the director of the Central Epidemic Command Center (CECC)—the two most popular misquoted targets—showed up in 975 (46.5%) and 1117 (53.3%) messages, respectively. Efforts were made to emphasize the authoritativeness of the quoted party as well. For example, titles for Zhong Nan-Shan became longer, from “Expert in Pandemic from Mainland China” and “Expert in Coronavirus” to “Expert in Coronavirus from Mainland China, 78-year-old Academian Zhong Nan-Shan.” Starting from April 1, 2020, every reported message had Zhong replaced with Chen Shih-Chung (Figure 10). As the Minister of the Ministry of Health and Welfare (MOHW) and director of Taiwan’s CECC, Chen’s popularity skyrocketed during the pandemic through his daily press conferences.

Due to the prevalence of this message spreading on the web and closed platforms, the MOHW and the CECC both sent out a press release [31] on April 2, 2020, reminding the public that this was misinformation. Nevertheless, this did not stop another viral spread of the same message at the end of a 4-day long weekend holiday in Taiwan, where crowds were seen at every tourist attraction on the island. For days, people worried that the long weekend would lead to another outbreak of the pandemic, providing an explanation as to why the message bearing the key topic “do not go out” would become a big hit.

#### Case 2: Drinking Salt Water Can Prevent the Spread of COVID-19

**Figure 11:**
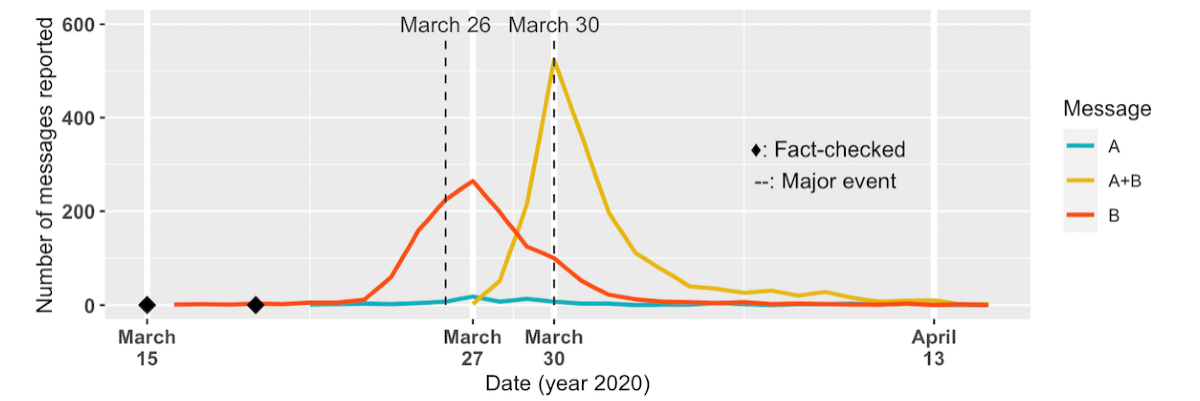
The number of Case 2 (ie, "Drinking salt water can prevent the spread of COVID-19") messages reported by date. The rumor had been fact-checked rather early; however, the information still received widespread attention. Message B peaked on March 27 with 265 messages, and the combined message peaked on March 30 with 523 messages. Refer to Table 5 for major societal events. Refer to Table 7 for contents of Message A, B, and A+B.

This rumor promoted drinking salt water to prevent COVID-19. Interestingly, this rumor was actually the combination of two individual rumors (Table 7). Message B had a peak on March 27 with 265 messages, and Message A+B received the most attention on March 30 with 523 messages (Figure 11).

**Table 7:** Content of Case 2 rumor.

Message	English translation	Original content
A	This is 100% accurate...Why did we see a huge decline of confirmed cases in China during the last few days? They simply forced their citizens to rinse mouths with salted water three times a day and then drink water for 5 minutes. The virus would attack throats before the lungs, and when getting in touch with salted water, the virus would die or get destroyed in lungs. This is the only way to prevent the spread of COVID-19. There is no need to buy medicine as there is nothing effective on the market.	這是100%準確的信息... 為什麼中國過去幾天大大減少了感染人數？他們只是簡單地強迫他們的人民每天漱口3次鹽水.完成後，喝水5分鐘.因為該病毒只能在喉嚨中侵襲，然後再侵襲肺部，當受到鹽水侵襲時，該病毒會死亡或從胃中流下來並在胃中銷毀，這是預防冠狀病毒流行的唯一方法.市場上沒有藥品，所以不要購買.
B	Before reaching the lungs, the novel coronavirus would survive in throats for 4 days. At this stage, people would experience sore throats and start coughing. If one can drink as much warm water with salt and vinegar as they can, the virus could be destroyed. Share this information to save people’s lives.	新冠肺炎在還沒有來到肺部之前，它會在喉嚨部位存活4天.在這個時候,人們會開始咳嗽及喉嚨痛.如果他能儘量喝多溫開水及鹽巴或醋,就能消滅病菌.儘快把此訊息轉達一下，因爲你會救他人一命！
A+B	Why did Mainland China show a huge decline of confirmed cases over the last few days? Besides wearing masks and washing hands, they simply rinse mouths with salted water three times a day and then drink water for 5 minutes...Dr Wang of Tung Hospital stated that the novel coronavirus would survive in throats for 4 days before reaching the lungs...If one can drink as much warm water with salt and vinegar as they can, the virus could be destroyed...	為什麼中國大陸過去幾天大大減少了感染人數？除了戴口罩勤洗手外，他們只是簡單地每天漱口3次鹽水.完成後，喝水5分鐘... 新冠肺炎在還沒有來到肺部之前，它會在喉嚨部位存活4天... 如果他能儘量喝多溫開水及鹽巴或醋，就能消滅病菌...

Among 3283 reported messages, 3093 (94.2%) misquoted medical professionals. The most popular misquoted parties were Dr Wang of Tung Hospital and the director of the Veteran Hospital*,* each seen in 2340 (71.3%) and 753 (22.9%) messages, respectively.

Drinking salt water to prevent COVID-19 was a popular false claim about COVID-19 internationally. This rumor was fact-checked several times in March by Taiwan’s fact-checking agencies [32,33], and even the WHO had fact-checked a similar claim about rinsing noses with saline [34]. However, this did not stop this piece of misinformation from receiving attention (Figure 11). In fact, several translations of the combined rumor (ie, Message A+B) were observed in April. The translations included English, Indonesian, Filipino, and Tibetan.

The lifespan of this “drink salt water” rumor was rather long. One famous fact-checking platform in Taiwan, MyGoPen, released an article to disprove this false medical advice again in October 2020 [35], 7 months after it was first seen in our data set.

#### Case 3: This Is a Critical Period; Here Are Some Suggestions

**Figure 12:**
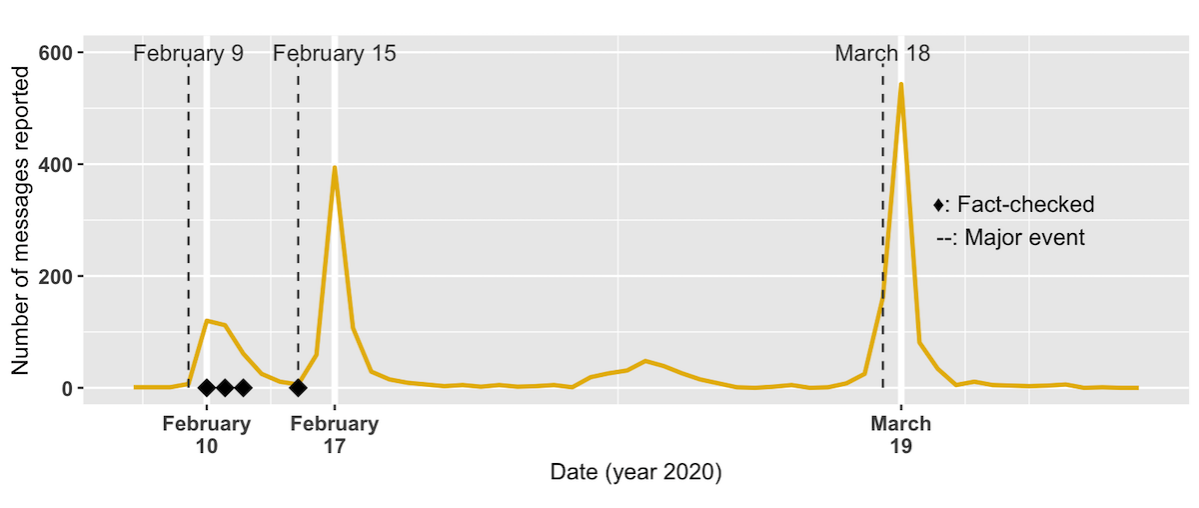
The number of Case 3 (ie, "This is a critical period; here are some suggestions") messages reported by date. The rumor was fact-checked several times in early February. However, higher peaks were still seen later on February 17 with 394 messages and, after a month, on March 19 with 543 messages. Refer to Table 5 for major societal events.

This rumor mentioned that Taiwan “entered a critical period of the pandemic” and provided a list of suggested measures for people to follow (Textbox 4). Some of the suggestions made sense in terms of personal hygiene, while others were without basis. This rumor first appeared in the data set on February 6 and included a total of 2121 messages. Over the 1.5 months of its most popular period, it went viral at least three times: February 10 with 120 messages, February 17 with 394 messages, and March 19 with 543 messages (Figure 12).

**Content of the Case 3 rumor.**

English translation:

10 days from now, Taiwan will be in a critical period to combat COVID-19. Here are some suggested measures.

1. Strictly prohibit going to public places. 2. Take out from restaurants. 3. Eat outside in open spaces. 4. Wash your hands the right way (extremely important). 5. When taking the subway or bus, choose the seats at the front half of the vehicle. 6. Do not wear contact lenses. 7. Eat warm food and more vegetables. 8. Avoid constipation. 9. Drink warm water. 10. Do not visit hair salons. 11. Hang the clothes you’re wearing outside for two hours the first moment you get home. 12. Do not wear jewelry. 13. Wash your hands immediately after touching cash or coins. Put coins you just received inside a plastic bag for one day before using them. 14. Do not use a colleague’s phone when working. If you have to, disinfect before using. 15. Avoid taking public transportation during rush hour. 16. Do not visit night markets or traditional markets. 17. Exercise. 18. Avoid going to the gym.

Original content:

今天開始10天，台灣正式進入武漢肺炎関鍵期。建議如下: 1.嚴禁進入公共場所. 2.用餐儘量將食物外帶.3.用餐環境儘量在外. 4.正確方式的洗手(特別重要). 5.坐捷運(公車)，選擇在車前頭. 6.避免戴隱形眼鏡 7.吃熱食,避開生凉食物,多吃蔬菜 8.保持腸胃暢通. 9.多喝溫水. 10.暫停去髮廊. 11.穿過的衣服(外套,長褲),回家先單獨吊在外2小時 12.暫停戴首飾. 13. 一有接觸錢幣,一定要洗手,剛拿進來的錢幣,先單獨放在塑膠袋中,一天後,才拿出來. 14.在公司不要使用別人的電話筒.電話筒需消毒.15. 避開巔峰時間坐車. 16.不去傳統市場及夜市. 17.適當的運動.18.暫停進入健身房.

**Figure 13:**
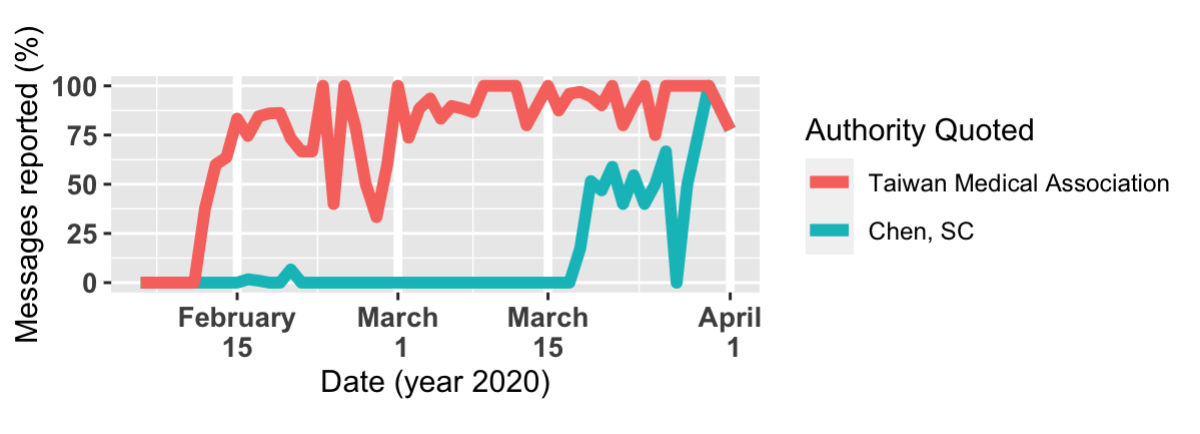
The Taiwan Medical Association (TMA) was quoted in almost every message in this rumor group, even though the TMA released a statement on February 12, 2020, saying that they did not endorse the material. Later, after Chen Shih-Chung went to Legislative Yuan on March 18, the same rumor started misquoting him.

Among 2121 reported messages, 1778 (83.8%) misquoted authorities as *endorsing* the rumor. The Taiwan Medical Association and the CECC director, Chen Shih-Chung, were the most misquoted parties, each seen in 1637 (77.2%) and 393 (18.5%) messages, respectively (Figure 13). A major revision of the rumor appeared on February 12 (Table 8), 6 days after the first message. In the revision, the original 18 bullets were pruned to 14, removing the ones that were perhaps more ridiculous or hard to follow. Strong words were also modified to a gentler tone. The Taiwan Medical Association, the most misquoted party, also first appeared in the message.

**Table 8:** Change log for the Case 3 rumor content.

Date	English translation (original)	
	Previous content	New content
**February 12, 2020**		
	Strictly prohibit going to public places. (嚴禁進入公共場所.)	Reduce going to public places. (減少進入公共場所.)
	Eat outside in open spaces. (用餐環境儘量在外.) When taking the subway or bus, choose the seats at the front half of the vehicle. (坐捷運(公車)，選擇在車前頭.) Do not visit hair salons. (暫停去髮廊.) Do not visit night markets or traditional markets. (不去傳統市場及夜市.)	Content was deleted
	No previous content	Regards from the Taiwan Medical Association. (醫師全聯會關心您.)
**March 18, 2020**		
	10 days from now, Taiwan will be in a critical period to combat COVID-19. Here are some suggested measures... (今天起10天，台灣正式進入武漢肺炎関鍵期，建議如下...)	10 days from now, Taiwan will be in a critical period to combat COVID-19 (explained by Chen Shih-Chung in the Legislative Yuan on March 18, 2020). Here are some suggested measures... (今天起10天，台灣正式進入武漢肺炎関鍵期，(3/18陳時中立法院說明) 建議如下...)
	Regards from the Taiwan Medical Association. (醫師全聯會關心您.)	Content was deleted

After almost a month with only a few messages circulating (Figure 12), on March 18, the CECC director, Chen Shih-Chung, went to the Legislative Yuan (similar to the US Congress) for interpellation about the pandemic. Chen started to be quoted in messages on the same day, making the “suggested measures” look like they were said by Chen during his interpellation (Table 8). The next day, on March 19, the reported message count skyrocketed to the highest peak. Of the 543 messages reported on March 19, 280 (51.2%) misquoted Chen.

Several fact-checking agencies published reports pointing out the falsity of the message [36-38] between February 10 and 15 (Figure 12). The Taiwan Medical Association, which was misquoted in 1637 out of 2121 (77.2%) messages, also released a clarifying statement on February 12 [39], stating explicitly that they did not endorse the material. However, similar to what we observed in the previous two cases, such fact-checking efforts did not prevent the rumor from getting widespread attention later. Rather, societal events might have played a larger role in the popularity of the rumor. For example, the spike on February 17 (Figure 12) was preceded by the first COVID-19 death case and a local cluster in Taiwan. A taxi driver tested positive for the virus and died the same day on February 15. Over the next few days, four of the driver’s family members also tested positive, forming the first local cluster of COVID-19 infection in Taiwan. The highest spike on March 19 was preceded by the CECC director’s interpellation in the Legislative Yuan, the event after which the messages started misquoting the director.

## Discussion

### Principal Findings

First, we demonstrated that by using a combination of HAC and KNN algorithms, we could efficiently separate a large number of social media text messages into fine-grained narratives, or *rumors*. The addition of the KNN classification algorithm enabled the speedup and, at the same time, achieved near-equivalent results compared to using HAC alone. Hence, this classification-based clustering algorithm could enable future large-scale studies of rumor transformation with social media post content.

We identified 396 rumors related to COVID-19 from the pool of 114,124 suspicious messages collected from the LINE platform between January and July 2020. Among the COVID-19–related rumors, more than one-third were deemed false or misleading by IFCN-certified fact-checking agencies in Taiwan. Compared to non–COVID-19–related messages, COVID-19–related messages were more likely to contain simplified Chinese characters or phrases originating from China. The association was statistically significant. As the official language in Taiwan is traditional Chinese, the result suggested that COVID-19–related messages were more likely to have originated from non-Taiwanese users than the non–COVID-19–related messages.

We qualitatively investigated three COVID-19–related rumors with the highest number of messages and observed several commonalities among these highly popular rumors. First, a significant number of messages from all three rumor groups misquoted key authoritative figures. Given the nature of the pandemic, the authorities were usually medical personnel. At times, a change in the quoted authority figures signaled a paradigm shift, indicating whom the public looked up to, for example, from Zhong Nan-Shan to Chen Shih-Chung. At other times, the quoted party did not seem to make any sense. For example, Dr Wang in Case 2 was in fact an orthopedist, a specialty not directly related to COVID-19. Second, in all three rumors, we observed spikes in reported messages even after several fact-checking agencies released reports that deemed the content false or misleading. Echoing the findings of Wood and Porter [40], the current practice of fact-checking did not seem to effectively stop the false information from getting widespread attention later. In fact, by identifying major societal events preceding each resurfacing peak, we asserted that resurfacing patterns were more influenced by major societal events and textual transformation. However, each peak of popularity would not last long, and it was often without good explanation about how one wave of attention ended.

Our work offers several insights into the landscape of misinformation in a closed platform as well as the behaviors of some popular COVID-19 rumors. These characteristics could serve as rules to discover possible false information as early detection mechanisms. Although we identified these characteristics manually in this study, it is quite possible to employ techniques such as NLP to automatically recognize these textual changes in the future, making it possible to have an automatic early warning system of possible misinformation before fact-checking efforts by professionals.

### Comparison With Prior Work

Our work adds to the limited collection of COVID-19 infodemic studies in closed platforms [41]. Compared with other rumor diffusion studies, such as the study of 17 political rumors by Shin et al [42], this work provided an efficient machine learning algorithm that could enable large-scale rumor evolution studies on social media platforms in the future. In comparison to other machine learning applications in COVID-19 infodemic studies, this work focused on fine-grained narratives, or *rumors*, rather than high-level topics, in order to study individual rumor propagation. To the best of our knowledge, this is the first study to examine rumor diffusion and propagation patterns of COVID-19 misinformation on a closed platform.

### Limitations

This study had several limitations. First, the data were collected by LINE users’ reports. Therefore, it was impossible to infer the true distribution of messages without making some assumptions. For example, if there was more health-related misinformation in our data, it did not necessarily translate to more health-related rumors circulating in the platform. In fact, it could also be that people were more alert and skeptical of health-related information. Second, we only looked at text messages. Therefore, information distributed visually or in audio form was not covered. Lastly, our algorithm for grouping messages does not work well with short texts.

### Conclusions

While social media may give rise to an unprecedented number of unverified rumors, it also provides a unique opportunity to study rumor propagation. In fact, to combat the infodemic, we need to first understand how and why some rumors became popular. In our studies, we proposed an algorithm that enables the research community to perform large-scale studies on the evolution of text messages at the rumor level rather than at the topic level. Moreover, we showed textual commonalities in widespread rumors in Taiwan during COVID-19. We also showed that the attention one rumor received was connected to major societal events and content changes. To the best of our knowledge, this is one of the few studies that has examined COVID-19 misinformation on a closed messaging platform and the first to examine the textual evolution of COVID-19–related rumors during their propagation. We hope that this will further spark more studies in rumor propagation patterns as an effort to fight the infodemic.

## Appendix

Multimedia Appendix 1 Experiment setup for comparing algorithms.

Multimedia Appendix 2 An example message without any of the COVID-19 keywords, but that could be identified as COVID-19–related by a close neighbor.

## Abbreviations

CECC: Central Epidemic Command Center
HAC: hierarchical agglomerative clustering
IFCN: International Fact-Checking Network
KNN: k-nearest neighbors
LDA: latent Dirichlet allocation
MOHW: Ministry of Health and Welfare
NLP: natural language processing
TCS: Taiwan Communication Survey
VADER: Valence Aware Dictionary and Sentiment Reasoner
WHO: World Health Organization
XGBoost: Extreme Gradient Boosting
