# Supplementary material for: The Evolution of Rumors on a Closed Social Networking Platform During COVID-19: Algorithm Development and Content Study
Source: JMIR Med Inform. 2021 Nov 23;9(11):e30467. doi: 10.2196/30467 (PMC8612313; doi:10.2196/30467)
Supplement: Multimedia Appendix 1 [file medinform_v9i11e30467_app1.docx]

## Experiments Setup for comparing algorithms

### Models for comparisons

We compared speed and performances among 4 models:

1. Hierarchical Agglomerative Clustering (**HAC**).
2. The Classification-based Clustering Algorithm (**HAC+KNN**).
3. LDA
4. KMeans with PCA dimensionality reduction (**KMeans**).

The clustering results from HAC serve as the gold standard for other models to compare with. Throughout the experiments we used the distance threshold **λ** = 0.6. Both **LDA** and **KMeans** require a predefined number of groups, a requirement which does not really fit our use case. However, for the sake of comparison, we would use the number of groups outputted by the HAC model as the parameter of both models.

### Evaluation Metrics

We used the standard precision, recall and F-score as evaluation metrics. In the sense of information retrieval, precision is the number of correct results returned divided by all results returned from search. Hence, high precision means the predictions are very relevant. On the other hand, *recall* measures the number of correct results returned divided by the total number of correct results. High recall corresponds to the completeness of returned results. Note that simply by returning all documents, one could achieve 100% of recall, but that will result in very low precision. Therefore, precision and recall need to be taken together to determine the quality of classification. F-score, defined as the harmonic mean of precision and recall, is one such measure that combines precision and recall.

### Evaluation Process

Suppose the input is a tokenized set of *k* documents *D^T^* and the **HAC** model puts *k* documents into *n* groups, (*g*_1_, *g*_2_, ... *g_n_*). *g*_1_ is the group having the largest number of documents and *g_n_* the least. Another model *M* puts *D^T^* into *m* groups: (*l*_1_, *l*_2_, ..., *l_m_*). We calculated precision, recall and F-score of model *M* by the algorithm in (Textbox S1).

In each experiment, we did 5 iterations. In each iteration, we randomly selected *k* messages from our dataset. We would get 1 precision, recall and F-score after each iteration, and we used the results of 5 iterations to calculate 95% confidence intervals.

Textbox S1. Algorithm for calculating Precision, Recall and F-score.

| ***Initialization:*** *i* ← 1, *c* ← 0, *p* ← 0, *r* ← 0, *f* ← 0; | |
| --- | --- |
| **while** c < $\frac{k}{2}$**do** | |
|  | Find *l_k_* where *l_k_* has the most overlapping components with *g_i_*;  calculate precision *p_k_*, recall *r_k_* and F-score *f_k_* of *l_k_* by comparing with *g_i_*;  *r* ← *r* + *r_k_*;  *p* ← *p* +*p_k_*;  *f* ← *f* + *f_k_*;  *i* ← *i* + 1;  *c* ←*c*+\|*g_i_*\|; |
| **Result:** precision ← p/i; recall ← r/i F-score ← f/i; | |

### Implementation

We implemented the experiments using Python 3.7 with package *gensim* for the LDA model, and *scikit-learn* for others.
