# Supplementary material for: The Evolution of Rumors on a Closed Social Networking Platform During COVID-19: Algorithm Development and Content Study
Source: JMIR Med Inform. 2021 Nov 23;9(11):e30467. doi: 10.2196/30467 (PMC8612313; doi:10.2196/30467)
Supplement: Multimedia Appendix 2 [file medinform_v9i11e30467_app2.docx]

|  | Original Text Message | English Translation |
| --- | --- | --- |
| Message containing COVID-related keyword. | 彰化計程車司機感染武漢肺炎死了！  底下幾個地方，據說這是他曾經去過的地方！  1. 彰化蔡文瑞診所  3. 金馬路車麗屋  3. 彰化751計程車  4. 楊漢銘醫院  5. 大富豪理容KTV  6. 全國理容KTV  7. 秀傳醫院  請大家留意....  多一分防範，少一分危險！ | A taxi driver in Changhua county died after testing positive for COVID-19!  It is said that he had visited the following places.   1. Changhua Tsai Clinic 2. Car Quality Automotive store in JinMa Road. 3. 751 Taxi Shop 4. Yang Hospital 5. The Rich KTV 6. The National KTV 7. Show Chwan Hospital   Please take care of yourself. |
| Message in the same rumor group with the previous message but does not contain any COVID-related keyword. | 彰化秀傳過世的人去過這些，  然後車麗屋員工已經發燒了  請大家小心  彰化蔡文瑞診所  金馬路車麗屋  彰化7510000計程車  楊漢銘醫院  大富豪理容KTV  全國理容KTV  秀傳醫院  這些都別去 | The person who died in Changhua Show Chwan Hospital had been to the following places. One staff member in the Car Quality Automotive store has had a fever.  Please take care of yourself.  Changhua Tsai Clinic  Car Quality Automotive store in JinMa Road.  7510000 Taxi Shop  Yang Hospital  The Rich KTV  The National KTV  Show Chwan Hospital  Do not go to the above places! |
